# Loss of YhcB results in overactive fatty acid biosynthesis

**DOI:** 10.1128/mbio.00790-24

**Published:** 2024-05-14

**Authors:** Hannah M. Stanley, M. Stephen Trent

**Affiliations:** 1 Department of Microbiology, College of Art and Sciences, University of Georgia, Athens, Georgia, USA; 2 Department of Infectious Diseases, College of Veterinary Medicine, University of Georgia, Athens, Georgia, USA; New York University School of Medicine, New York, New York, USA

**Keywords:** fatty acids, YhcB, phospholipids, outer membrane, lipopolysaccharide, Mla, PldA, ACC, acetyl-CoA carboxylase complex, AccABCD, lpxM, glycerophospholipids

## Abstract

Loss of the *Escherichia coli* inner membrane protein YhcB results in pleomorphic cell morphology and clear growth defects. Prior work suggested that YhcB was directly involved in cell division or peptidoglycan assembly. We found that loss of YhcB is detrimental in genetic backgrounds in which lipopolysaccharide (LPS) or glycerophospholipid (GPL) synthesis is altered. The growth defect of Δ*yhcB* could be rescued through inactivation of the Mla pathway, a system responsible for the retrograde transport of GPLs that are mislocalized to the outer leaflet of the outer membrane. Interestingly, this rescue was dependent upon the outer membrane phospholipase PldA that cleaves GPLs at the bacterial surface. Since the freed fatty acids resulting from PldA activity serve as a signal to the cell to increase LPS synthesis, this result suggested that outer membrane lipids are imbalanced in Δ*yhcB*. Mutations that arose in Δ*yhcB* populations during two independent suppressor screens were in genes encoding subunits of the acetyl coenzyme A carboxylase complex, which initiates fatty acid biosynthesis (FAB). These mutations fully restored cell morphology and reduced GPL levels, which were increased compared to wild-type bacteria. Growth of Δ*yhcB* with the FAB-targeting antibiotic cerulenin also increased cellular fitness. Furthermore, genetic manipulation of FAB and lipid biosynthesis showed that decreasing FAB rescued Δ*yhcB* filamentation, whereas increasing LPS alone could not. Altogether, these results suggest that YhcB may play a pivotal role in regulating FAB and, in turn, impact cell envelope assembly and cell division.

**IMPORTANCE:**

Synthesis of the Gram-negative cell envelope is a dynamic and complex process that entails careful coordination of many biosynthetic pathways. The inner and outer membranes are composed of molecules that are energy intensive to synthesize, and, accordingly, these synthetic pathways are under tight regulation. The robust nature of the Gram-negative outer membrane renders it naturally impermeable to many antibiotics and therefore a target of interest for antimicrobial design. Our data indicate that when the inner membrane protein YhcB is absent in *Escherichia coli*, the pathway for generating fatty acid substrates needed for all membrane lipid synthesis is dysregulated which leads to increased membrane material. These findings suggest a potentially novel regulatory mechanism for controlling the rate of fatty acid biosynthesis.

## INTRODUCTION

The bacterial Gram-negative outer membrane (OM) acts as a selectively permeable barrier, allowing the cell to adapt to a wide range of environments and stresses. A key feature of this barrier is that it is asymmetrical with glycerophospholipids (GPLs) in the inner leaflet and primarily lipopolysaccharide (LPS) in the outer leaflet (1). The OM is also tightly associated with the peptidoglycan cell wall that is located in the periplasm. This region of space is between the OM and the cytoplasmic inner membrane (IM), which is composed of only GPLs (1).

Fatty acids serve as the building blocks for numerous lipids that are critical for generating amphipathic membranes. In *Escherichia coli*, fatty acids are obtained via exogenous and endogenous means, either imported from the environment or generated through fatty acid biosynthesis (FAB) (2). This biosynthesis pathway in *E. coli* is a type II FAB system, being composed of independent enzymes that together coordinate synthesis (2). The majority of fatty acids generated by FAB (acyl-acyl carrier protein [ACP]) are used in GPL and LPS synthesis with a large proportion going to GPLs. Exogenous fatty acids can also be converted into acyl-coenzyme A (CoA) and used in GPL synthesis or degraded via β-oxidation for energy. However, with few exceptions (3), acyl-CoAs cannot be used in LPS synthesis because the acyltransferases in this pathway utilize only acyl-ACPs as substrates (4). Therefore, construction of the OM requires cooperation of the complex LPS and GPL biosynthetic pathways with regard to both sharing fatty acid pools and coordinating membrane components.

**Fig 1:**
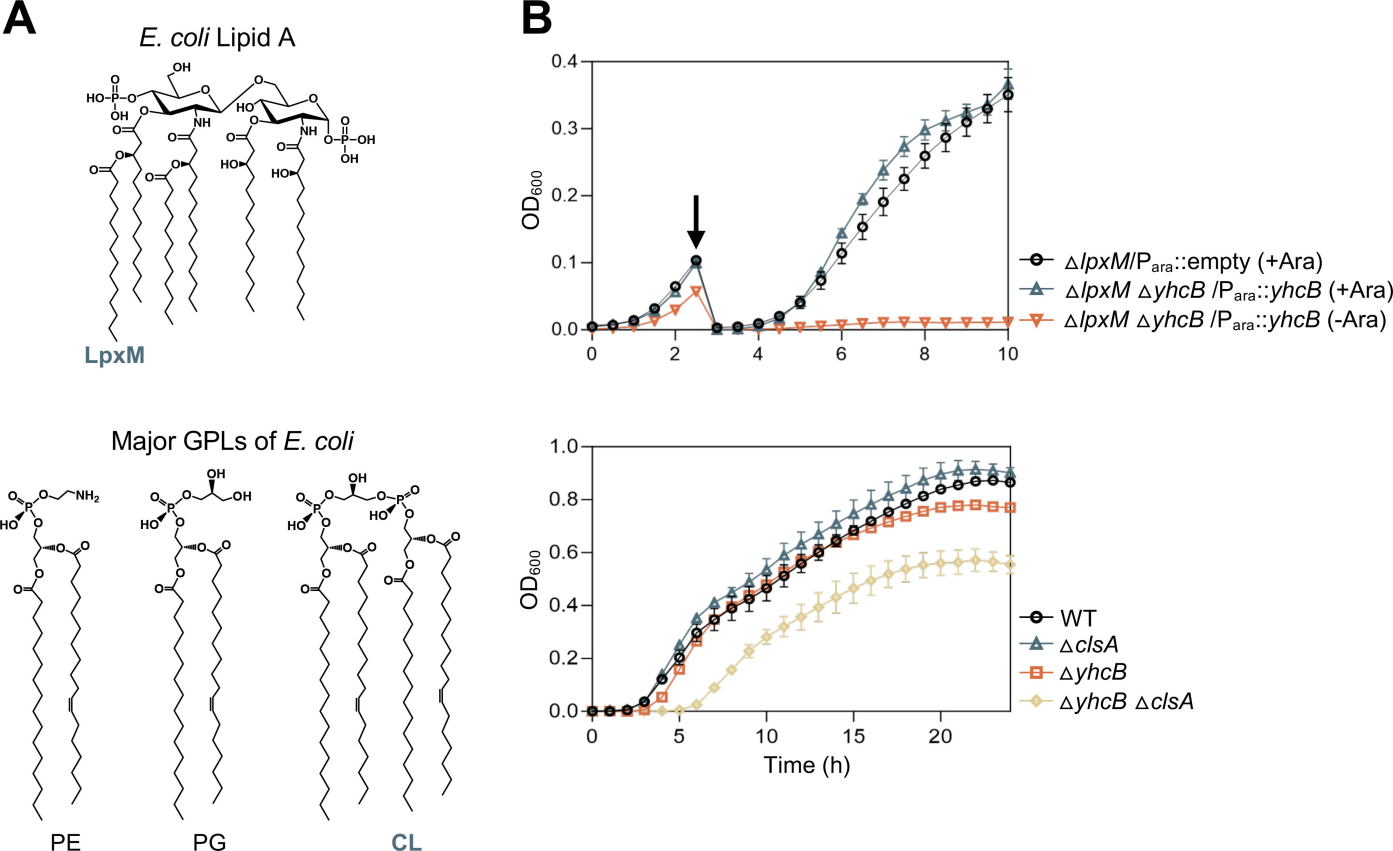
Depletion of YhcB in an *lpxM yhcB* mutant is lethal and a *yhcB clsA* mutant is synthetically sick. (**A**) Chemical structure of the lipid A domain of LPS (top). The acyl chain added by LpxM is indicated. The structures of the major GPLs (phosphatidylethanolamine [PE], phosphatidylglycerol [PG], and cardiolipin [CL]) found in *E. coli* are shown (bottom). (**B**) Top panel: the strain Δ*lpxM* Δ*yhcB* + P*_ara_::yhcB* was grown under inducing conditions with 0.1% arabinose (blue) or under repressing conditions with 0.2% glucose (orange). Black arrow denotes when strains were diluted 1:100. The Δ*lpxM* + P*_ara_*::empty strain (black) was used as a control. Bottom panel: wild type, Δ*clsA*, Δ*yhcB*, and Δ*yhcB* Δ*clsA* were grown for 24 hours. For both panels, cultures were grown at 37°C in a 96-well plate, and OD_600_ was assessed every 30 minutes using a microplate reader. Data shown are representative of biological triplicates.

LPS synthesis begins with the generation of its lipid anchor, referred to as lipid A, followed by the addition of the core-oligosaccharide and the O-antigen domain. The lipid A-core domains are synthesized on the cytoplasmic side of the IM, and, when present, O-antigen addition occurs in the periplasm. Nine enzymes are involved in synthesizing the hexa-acylated lipid A anchor of *E. coli* (4, 5). Full acylation of the molecule is important for LPS transport across the cell envelope and for OM stability. For example, the final acyltransferase LpxM (Fig. 1A) is not required for cell viability, but *lpxM* mutants producing penta-acylated LPS show a reduction in LPS transport, a decrease in LPS synthesis, and an increased sensitivity to antibiotics (6, 7).

The major GPLs of *E. coli* [(phosphatidylethanolamine [PE], phosphatidylglycerol [PG], and cardiolipin [CL]) (Fig. 1A) are also assembled at the cytoplasmic surface of the IM and are generated using the universal GPL precursor, phosphatidic acid (1). Enzymes PlsB and PlsX/Y generate lysophosphatidic acid which PlsC uses to synthesize phosphatidic acid. While lysophosphatidic acid synthesis is redundant, PlsB is the dominant generator of the lipid and is essential for growth (8). The two main GPL species (PE and PG) are then synthesized through two branching pathways with CL being generated through the condensation of two PG molecules or a single PE and PG. Interestingly, PE and PG are the only essential GPLs in *E. coli*. Cardiolipin is dispensable to wild-type *E. coli* despite three CL synthases encoded in the genome (9).

While studying how LPS and GPL syntheses are coordinated for optimal assembly of the cell envelope, Douglass et al. (7) published transposon sequencing (TnSeq) data sets for *lpxM*-null and *clsA*-null mutants. Loss of the LPS acyltransferase LpxM was found to be synthetically lethal with deletion of *clsA*, the gene encoding the major CL synthase. Ultimately, CL was shown to be critical for proper LPS transport, further demonstrating how LPS and GPL syntheses are connected (7). Interestingly, in the separate *lpxM* and *clsA* knockout mutants, a gene encoding the protein YhcB was also predicted to be necessary for fitness (7). At the time the current study was initiated, little was known about the role of YhcB in the cell.

YhcB is categorized as a non-essential IM protein, possessing one transmembrane domain and three α-helical domains that are localized within the cytoplasm. It is predicted to form a homo-oligomeric complex of approximately 5–12 subunits based upon blue native-/SDS-PAGE analysis (10), though a crystal structure of YhcB from *Haemophilus ducreyi* suggests that it might form a tetramer (11). The loss of *yhcB* in *E. coli* results in antibiotic sensitivity, aberrant cell morphology, and a growth defect (11–13). Niba and co-authors (14) first reported that deletion of *yhcB* is synthetically lethal in cells lacking RodZ, an IM protein involved in determining rod cell shape (15). In a follow-up study, the group (16) investigated whether YhcB interacts with key peptidoglycan synthesis, cytoskeleton, and cell division proteins through bacterial two-hybrid system experiments. They proposed that YhcB interacts with not only RodZ but also cell shape protein MreCD, peptidoglycan synthesis proteins RodA and MurG, and a protein implicated in the regulation of LPS synthesis LapA (16, 17). The authors suggested these interactions indicate that YhcB facilitates the bridge between peptidoglycan synthesis and cell shape maintenance. Notably, these potential YhcB interaction partners were not verified with additional experiments.

Due to a potential yet unverified interaction with RodZ, YhcB was examined as a cell division protein by visualizing Z-ring formation. Loss of YhcB resulted in mislocalized FtsZ, and the authors concluded that YhcB is involved in cell division (11). However, YhcB has never been shown to directly interact with FtsZ or co-localize with any proteins required for Z-ring assembly. Furthermore, it is important to note that disrupting the synthesis or transport of LPS or GPLs can also result in cell division defects (7, 18–20).

A recent transposon mutagenesis screen of a *yhcB* mutant (13) indicated that perturbation of cell division machinery or disruption of genes impacting the synthesis of LPS, enterobacterial common antigen, or peptidoglycan reduces bacterial fitness and may lead to cell death. It is apparent that the loss of YhcB results in cell-wide dysregulation, and for that reason, several groups have concluded that YhcB must be involved in multiple biogenesis pathways (11, 13). However, the striking cellular defects arising from loss of YhcB confound broad-scale analysis.

It has been established in the literature that deletion of *yhcB* results in filamentous cells that are highly sensitive to envelope stresses (11–13). Increased antibiotic sensitivity can be the result of membrane permeability defects, often caused by disruption of OM asymmetry. Published TnSeq data from our laboratory (7) and others (13) show that *yhcB* becomes important when *lpxM* is chromosomally deleted, suggesting that YhcB may have a role in membrane synthesis. Our data support published accounts that show *yhcB* mutants are pleomorphic with severe fitness defects. Here, we find that cells lacking YhcB appear to aberrantly generate fatty acids which preferentially shuttle into GPL biosynthesis. This flux results in an imbalanced ratio of LPS and GPLs within the cell, resulting in major cell envelope defects. By manipulating FAB through genetic or chemical means, these defects can be mitigated and fitness of the *yhcB* mutant can be restored. We therefore conclude that YhcB may be involved in the regulation of FAB and that it may support cell envelope assembly and cell division through this pathway.

## RESULTS

### Deletion of *lpxM* or *clsA* in a *yhcB* mutant results in synthetic lethal and synthetic sick phenotypes, respectively

Random transposon (Tn) mutagenesis in individual *lpxM* and *clsA* mutants paired with next-generation sequencing (TnSeq) suggested that loss of *yhcB* would be detrimental to bacterial fitness in these strains (7). Using generalized transduction and the Keio strain collection (21), we attempted to generate a strain lacking *yhcB* in combination with either *lpxM* or *clsA*. In the case of the *lpxM* mutant, attempts to delete *yhcB* resulted in no transductants, and changing the order of deletion had no effect. To confirm a possible synthetic lethal phenotype, a plasmid-expressing *yhcB* from an arabinose-inducible promoter (P*_ara_::yhcB*) was introduced into Δ*lpxM* followed by deletion of *yhcB* by phage transduction in the presence of arabinose. The *lpxM yhcB* mutant, carrying either empty vector or P*_ara_::yhcB*, was grown in lysogeny broth (LB) with either an inducer or a repressor (glucose) (Fig. 1B). Growth arrests when YhcB is depleted in the *lpxM yhcB* double mutant, demonstrating synthetic lethality. In the case of *clsA*, we were able to generate the *yhcB clsA* double mutant. Compared to the *yhcB* single mutant, however, Δ*yhcB* Δ*clsA* exhibited a worsened growth defect compared to the parent strains, indicating a synthetic sick phenotype (Fig. 1B). These results suggested that when the membrane is already perturbed by the loss of LpxM or ClsA, the additional loss of YhcB weakens the cell envelope further. Given that LpxM and ClsA have related functions in LPS biosynthesis/transport, these phenotypes suggest a connection of YhcB to the cell envelope (7).

### Characterization of the *yhcB* mutant in W3110

All prior work investigating YhcB was performed in *E. coli* strains BW25113 and MG1655. It has been reported that many MG1655 laboratory stocks possess a 1-bp deletion in *glpR* that encodes the repressor of the glycerol-3-phosphate regulon, a mutation predicted to abolish function (22). Loss of this regulator leads to constitutive expression of glycerol metabolism genes, including genes involved in degradation of an important component used in GPL synthesis (23). The BW25113 strain, used as the parent strain for the Keio collection, possesses a Gly-to-Thr missense mutation in *fabR* and has been shown to act similarly to a Δ*fabR* mutant (21, 24). FabR is a repressor of *fabA* and *fabB* and therefore has a direct impact on FAB when disrupted (24). Because these two strains have mutations impacting key GPL precursors, we chose to work in K-12 strain W3110. This strain does not contain mutations that could affect how the cell adapts to dysregulation of fatty acid or GPL synthesis as confirmed by whole-genome sequencing of our laboratory W3110 stock (22, 24) (see Data Set S1 in supplemental material).

First, deletion of *yhcB* in W3110 was evaluated for changes in growth and cell shape. By both measuring optical density (OD_600_) and determining colony-forming units (CFU), it was clear that Δ*yhcB* has a drastic growth defect (see Fig. S1A in supplemental material). Scanning electron microscopy (SEM) of Δ*yhcB* showed the reported pleomorphic phenotype in the W3110 background (Fig. S1B). There were cells of increased length (more than 60 µm), cells with abnormal division sites, and cells displaying increased surface volume. We observed after 24 hours of liquid culture at 37°C that the *yhcB* mutant had visible cell debris in the culture tube (Fig. S1C). Loss of YhcB also rendered *E. coli* more sensitive to vancomycin, an antibiotic that does not easily penetrate the OM, suggesting changes in OM integrity (Fig. S1D).

### Suppressors that rescue synthetic lethality and growth defect

We sought to isolate suppressors that would provide insight into the function of YhcB in the cell. To do so, we used two methodologies: one that focused on what mutations would sustain viability in the synthetic lethal *yhcB lpxM* mutant, and the other focused on what mutations would restore normal growth in the single *yhcB* mutant.

**Fig 2:**
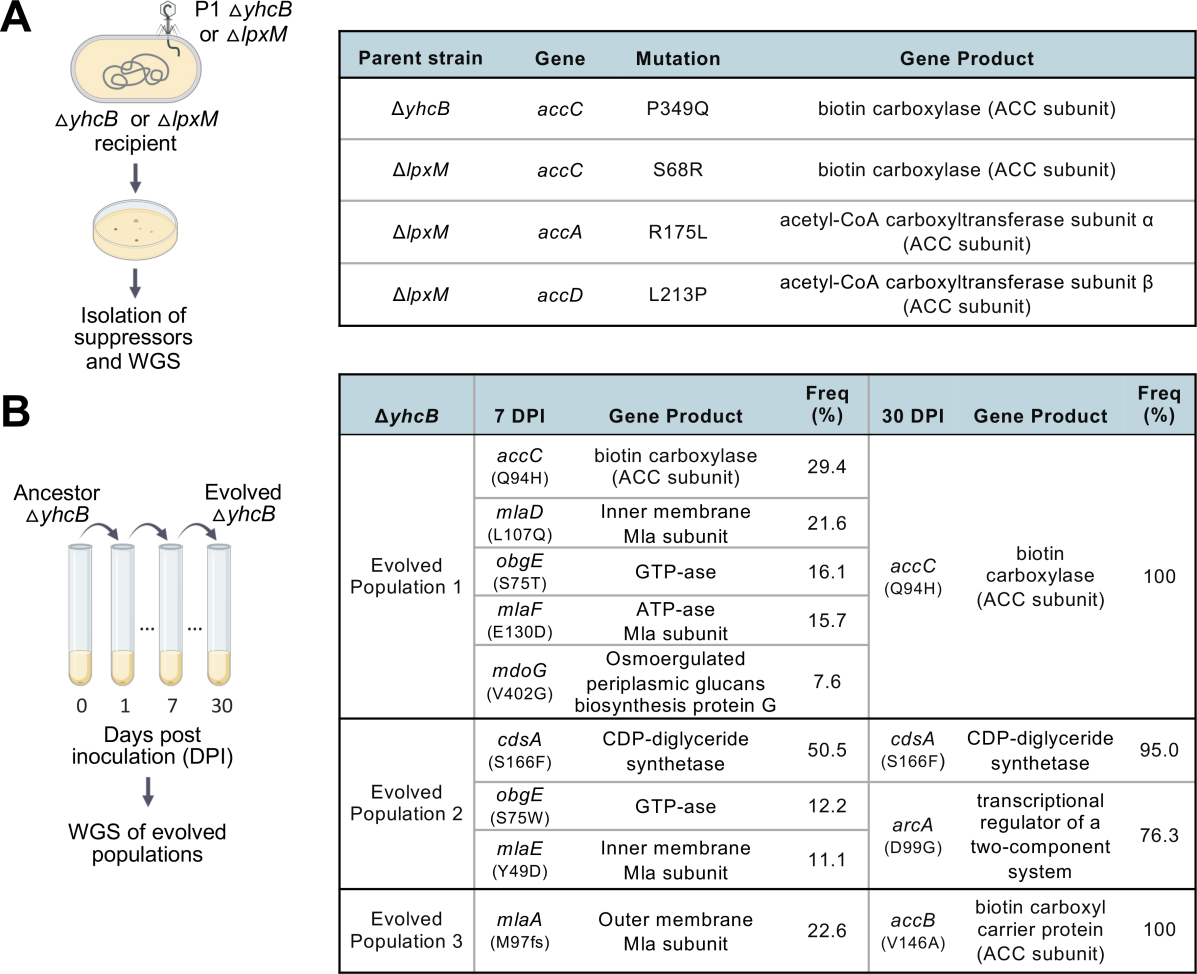
Summary of spontaneous suppressor mutations. (**A**) Suppressor mutations that restored viability of the synthetically lethal *yhcB lpxM* double deletion. (**B**) Summary of mutations that occurred during a short-term evolution experiment of three independent *yhcB* mutant populations. Bacteria were subjected to whole-genome sequencing at the time cellular debris was no longer observed (7 days) and at the end time point (30 days) of the experiment. DPI, days post inoculation. Created in part with BioRender.com.

Suppressors that restored viability of Δ*yhcB* Δ*lpxM* were obtained via large-scale transductions intended to isolate the infrequent successful transductants. Independent transductions were repeatedly performed for each single mutant, *yhcB* or *lpxM,* to chromosomally delete the secondary gene. Resulting colonies were PCR screened for successful deletions and then subjected to whole-genome sequencing (Fig. 2A). Multiple suppressor mutations arose within three of the four genes encoding the subunits of the acetyl-CoA carboxylase (ACC) complex, two suppressors in *accC,* and one in each *accA* and *accD*. The AccABCD complex initiates FAB and catalyzes the carboxylation of acetyl-CoA to form malonyl-CoA, an enzymatic product that is dedicated solely to FAB.

Suppressors that restored the growth defect of the *yhcB* mutant were obtained via a short-term evolution experiment. Three independent cultures were passaged daily until the cellular debris that is always present after 24 hours of growth of Δ*yhcB* was no longer visible (Fig. S1C). Surprisingly, all three independent populations were visually free of debris on day 7, and an aliquot of each was sent for whole-genome sequencing. The cultures were continued until day 30 when populations were whole-genome sequenced a second time (Fig. 2B).

At the time that visual cell debris had resolved, no one particular mutation had fully overtaken any of the populations. In population 1, mutations in *accC, mlaD, obgE, mlaF,* and *mdoG* were found at varying frequencies. These genes encode (i) the acetyl-CoA carboxylase subunit α (part of ACC complex), (ii) an IM subunit of the Mla (maintenance of OM lipid asymmetry) system involved in GPL transport, (iii) a GTPase implicated in stringent response, (iv) an ATPase of the Mla system, and (v) a periplasmic protein involved in the formation of osmoregulated periplasmic glucans, also known as membrane-derived oligosaccharides (Fig. 2B). After 30 days of continuous passaging, the only mutation in evolved population 1 was the original *accC* mutation found at day 7. Population 2 originally had mutations in *cdsA* (the CDP-diglyceride synthetase required for GPL synthesis), *obgE*, and *mlaE*, which encodes another component of the Mla system. After 30 days, the *cdsA* mutation had become the dominant mutation in population 2, and a mutation in *arcA* (the transcriptional regulator of a two-component system) had arisen. The third population only had a mutation in *mlaA* (the OM lipoprotein subunit in the Mla transport system) when cellular debris was no longer observed, and by day 30, a mutation in *accB* (the biotin carboxyl carrier protein in the ACC complex) had overtaken the population. All three populations still displayed filamentous cell morphology at day 7, despite visual debris resolution (Fig. S2). The recurring mutations in genes involved in FAB or GPL biosynthesis/transport suggested that YhcB may be involved in lipid biogenesis.

### Disruption of the Mla system rescues growth defect caused by loss of YhcB

**Fig 3:**
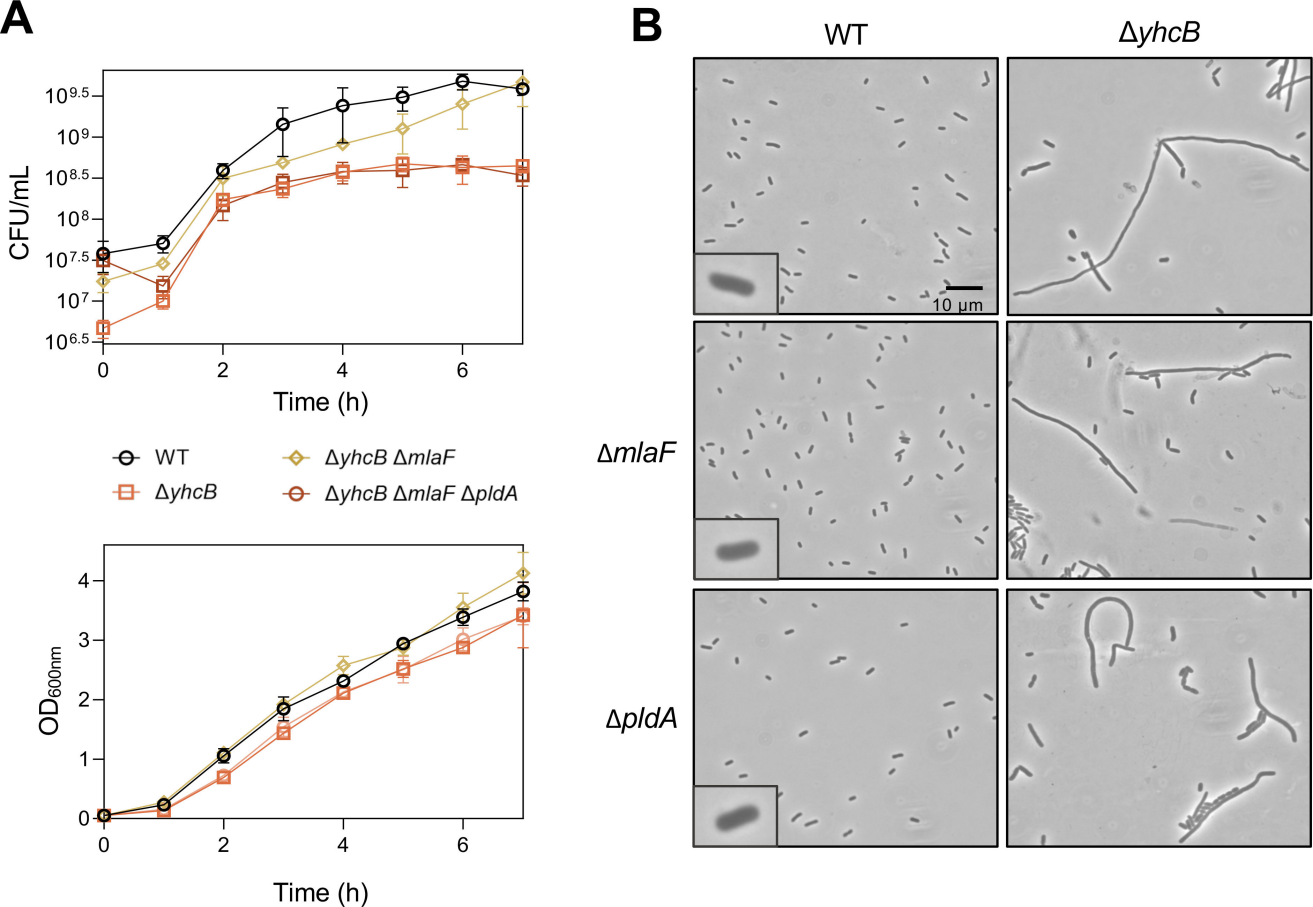
Loss of the Mla retrograde lipid transport system rescues the growth defect but not cell morphology of Δ*yhcB* in a PldA-dependent manner. (**A**) Growth was assessed in 5 mL LB and measured by both OD_600_ (bottom) and serial dilutions (top) to calculate CFU/mL. Data shown are representative of biological triplicates. (**B**) Bacteria represented in the growth curves were imaged using phase-contrast microscopy at ×1,000 magnification with a 10-µm scale bar that is indicated in the top left panel. Additional controls can be found in Fig. S3.

Because mutations in the Mla system were found in every evolved population when debris was resolved, we investigated the role of this system in the *yhcB* mutant. The Mla system carries out retrograde transport of mislocalized GPLs in the outer leaflet of the OM for reinsertion into the IM, helping to maintain the asymmetrical nature of the OM (25–27). Loss of any one of the six Mla proteins abolishes its retrograde transport of GPLs (28). To evaluate whether our *mla* mutations were disrupting function, we deleted *mlaF* (encodes the ATPase subunit of the ABC transporter complex) which was mutated in evolved Δ*yhcB* population 1. Deletion of *mlaF* improved cell survivability as evidenced by an increase in CFU over time compared to the Δ*yhcB* parent (Fig. 3A) This rescue of the growth defect suggests that the *mla* suppressors across all three evolved populations were loss-of-function mutations.

In addition to the Mla system, *E. coli* possesses an OM phospholipase, PldA, that helps maintain asymmetry of the OM by cleaving GPLs mislocalized to the cell surface. The fatty acids released by PldA activity act as a signal to increase LPS synthesis and restore the balance of OM lipids (29). Because it is known that abolishing Mla transport causes an increase in PldA activity due to accumulated GPLs in the OM, we deleted *pldA* from the *yhcB mlaF* mutant (28). When PldA function is lost, the growth defect returns (Fig. 3A; Fig. S3). This result indicates that the fitness improvement caused by the loss of Mla function is likely mediated by PldA and suggests that the LPS to GPL ratio in the *yhcB* mutant may be out of balance. A change in OM asymmetry, whether caused by too many GPLs or too little LPS, may also explain the synthetic phenotypes seen with *lpxM* and *clsA* deletions. Although the growth defect was resolved in the *yhcB mlaF* strain, it did not have improved morphology and remained filamentous (Fig. 3B). These mutations weakly suppressed the *yhcB* phenotypes by rescuing only the growth defect but were eventually overtaken by the FAB- and GPL-related mutations in the evolved populations.

### Reducing fatty acid biosynthesis rescues *yhcB* phenotypes

**Fig 4:**
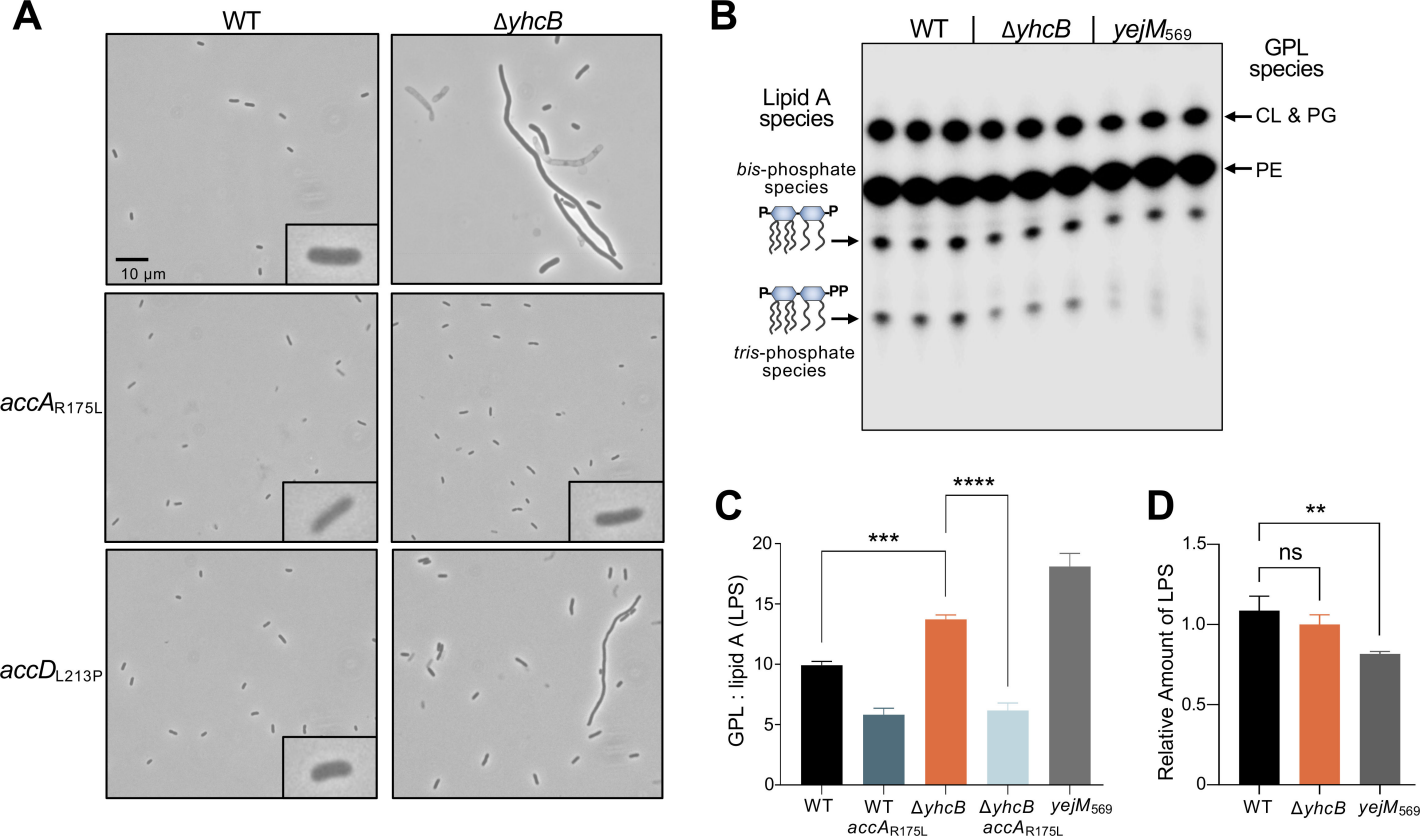
Mutations in the ACC complex rescue Δ*yhcB* cell morphology and restore the GPL:lipid A (LPS) ratio. (**A**) Alleles of *acc* (*accA*_R175L_ and *accD*_L213P_) that restored viability of a *yhcB, lpxM* double mutant were transduced into a single *yhcB* mutant and evaluated by phase contrast microscopy at ×1,000 magnification (10 µm scale bar). (**B**) Thin-layer chromatography (TLC) of total ^32^P-labeled lipids (both lipid A and GPLs) extracted from wild type, the *yhcB* mutant, and wild type with a truncated *yejM* allele (*yejM*_569_). The latter strain has greatly reduced LPS levels and serves as a control for an altered GPL:lipid A (LPS) ratio. Major species of GPLs are indicated on the right side of the TLC and major lipid A species on the left. Image shown has been adjusted for visibility of all lipid species; an unsaturated image was used for densitometry calculations. (**C**) Bar graph representing the GPL:lipid A (LPS) ratios of wild type, Δ*yhcB* with wild-type *accA*, and Δ*yhcB* with the *accA*_R175L_ allele. Again, *yejM*_569_ strain is included as a control. Data shown are representative of biological triplicates. Significance was assessed using two-tailed, unpaired *t*-tests. (**D**) Bar graph representing relative amounts of LPS of wild type, Δ*yhcB*, and *yejM*_569_ quantified using densitometry. Data are representative of biological triplicates. Significance was assessed using two-tailed, unpaired *t*-tests. NS indicates not significant; *, *P*  ≤  0.05; **, *P*  ≤  0.01; ***, *P*  ≤  0.001; ****, *P*  ≤  0.0001.

Multiple suppressor mutations in genes encoding the subunits of the ACC complex arose in the *yhcB lpxM* double mutant, allowing viability. To examine these mutations solely when YhcB function is lost, deletions were made in genes close to *accA* and *accD* (*yaeR* and *purF*, respectively). Taking advantage of the close genetic linkage, we then used P1 phage-mediated transduction to transfer the mutant alleles of *accA* and *accD* from the suppressor mutants into wild-type W3110 and Δ*yhcB*. These mutants were then assessed visually via microscopy. W3110 WT carrying either the *accA*_R175L_ or *accD*_L213P_ allele appeared slightly smaller than wild type, an expected phenotype if fatty acid production has decreased (see Fig. S4A and B). Introduction of *accA*_R175L_ into Δ*yhcB* resulted in strong suppression of the gross morphological defects seen in the parent. The *yhcB accD*_L213P_ strain had a partial recovery of the filamentous *yhcB* phenotype (Fig. 4A and Fig. S4). It is important to note deletion of either *yaeR* or *purF* for introduction of the mutant *acc* alleles had no effect on cell morphology (Fig. S4C).

Because both suppressor screens had suggested that FAB was connected to the *yhcB* defects, overall lipid content was analyzed from cells grown in the presence of ^32^P_i_. The freed lipid A domain of LPS and GPLs were isolated in the same extraction, and the lipid species were separated by thin-layer chromatography (TLC). In this solvent system, the major GPLs migrate faster than lipid A species, with PG and CL migrating to the same position. The *yhcB* mutant exhibits a growth defect that worsens in stationary phase (Fig. S1; Fig. 3A); therefore, lipids were extracted from cultures at that phase of growth to better assess what defects were contributing to the growth phenotype. As a control, we also included W3110 expressing a truncated variant of YejM (*yejM*_569_). LPS biosynthesis is negatively regulated by YejM, and LPS (lipid A) synthesis in this mutant is greatly reduced (30), resulting in a significant increase in the GPL:lipid A ratio (Fig. 4B and C). Similar to the *yejM* mutant, loss of YhcB resulted in a clear increase in the GPL:lipid A ratio. However, because this quantification measures the ratio of all GPL species compared to all lipid A species, reduced LPS *or* increased GPLs can result in an increased GPL:lipid A ratio as seen in both the *yejM* and the *yhcB* mutants.

To evaluate whether the increased GPL:lipid A (LPS) ratio in the *yhcB* mutant was due to a reduction in LPS synthesis, proteinase K-treated whole-cell lysates were subjected to SDS-PAGE, and the LPS was visualized by staining with Pro-Q Emerald carbohydrate dye. Deletion of *yhcB* did not result in a significant change in LPS production, whereas the *yejM_569_* control showed the expected reduction in LPS levels (Fig. 4D; Fig. S5A). Thus, the altered GPL:lipid A ratio observed in Δ*yhcB* must arise from an increase in GPLs. To answer whether the *accA*_R175L_ allele was fixing morphology by restoring lipid balance, total lipids were again extracted. The increased GPL:lipid A ratio of the *yhcB* mutant is abolished when the *accA*_R175L_ allele is introduced (Fig. 4C; Fig. S5B and C). Mirroring the cell morphology data, the *accD*_L213P_ allele only partially restored lipid levels (Fig. S5B and C). When the *acc* alleles are introduced, the *yhcB* phenotype is suppressed, suggesting that these mutations are epistatic to *yhcB* and that this rescue is upstream of the *yhcB* defect.

Because mutations that rescued viability of the *yhcB lpxM* double mutants suggested a connection to FAB, we next assessed how chemical inhibition of FAB would affect the *yhcB* mutant. Cerulenin is an inhibitor that prevents FAB elongation by irreversibly binding FabB and FabF (31–33). Several groups have suggested that the defects of *yhcB* are due to a potential role in cell division, so we included an *envC* knockout mutant as a control (11, 12). EnvC activates key peptidoglycan amidases that are required for septal division and exhibits a filamentation defect visually similar to the *yhcB* mutant (34).

**Fig 5:**
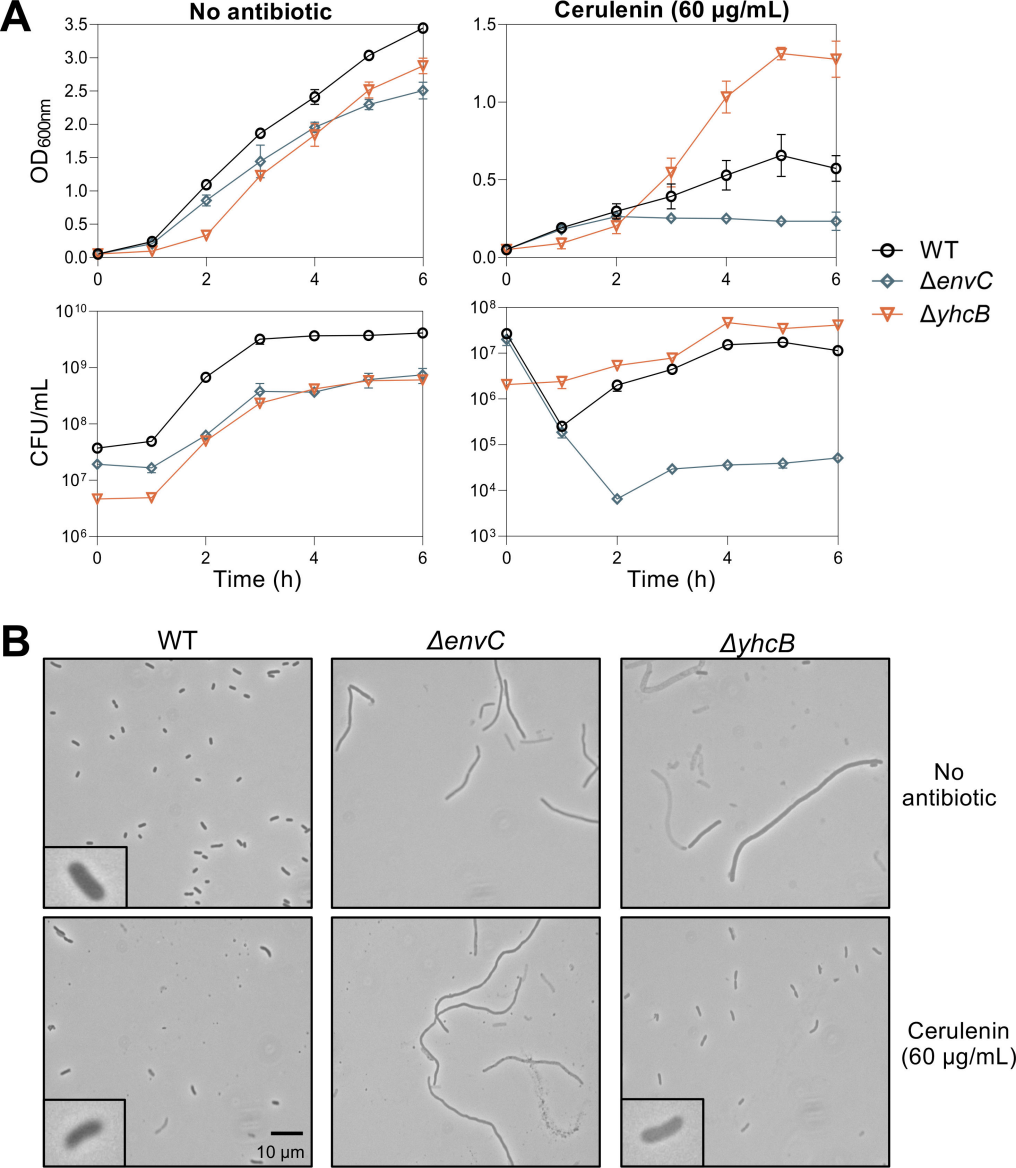
Chemical inhibition of FAB rescues Δ*yhcB* growth defect and cell morphology. (**A**) Growth was assessed by measuring OD_600_ (top panels) and by serial dilutions (bottom panels) to calculate CFU/mL every hour. Wild type, Δ*yhcB*, and Δ*envC* (control strain) were grown in LB with and without cerulenin (60 µg/mL). (**B**) Bacteria represented in the growth curves were imaged at hour 5 using phase-contrast microscopy at ×1,000 magnification with a 10-µm scale bar for all panels. Data shown are representative of biological triplicates.

As expected, growth of wild-type W3110 is inhibited at 60 µg/mL of cerulenin. Cells lacking EnvC showed increased sensitivity to the antibiotic, and inhibition of FAB had no impact on Δ*envC* morphology. Strikingly, the *yhcB* mutant grows well in the presence of cerulenin and better than wild type (Fig. 5A). Furthermore, the presence of the antibiotic restored cell morphology of Δ*yhcB* (Fig. 5B) and ameliorated the growth defect, acting as a strong suppressor of the *yhcB* phenotypes. The fact that cerulenin could not rescue a generalized cell division defect typified by Δ*envC* yet rescues the morphology of Δ*yhcB* suggests that the cause of the Δ*yhcB* morphological defect is related to FAB.

### The phenotypes associated with *yhcB* are due to excess fatty acids

**Fig 6:**
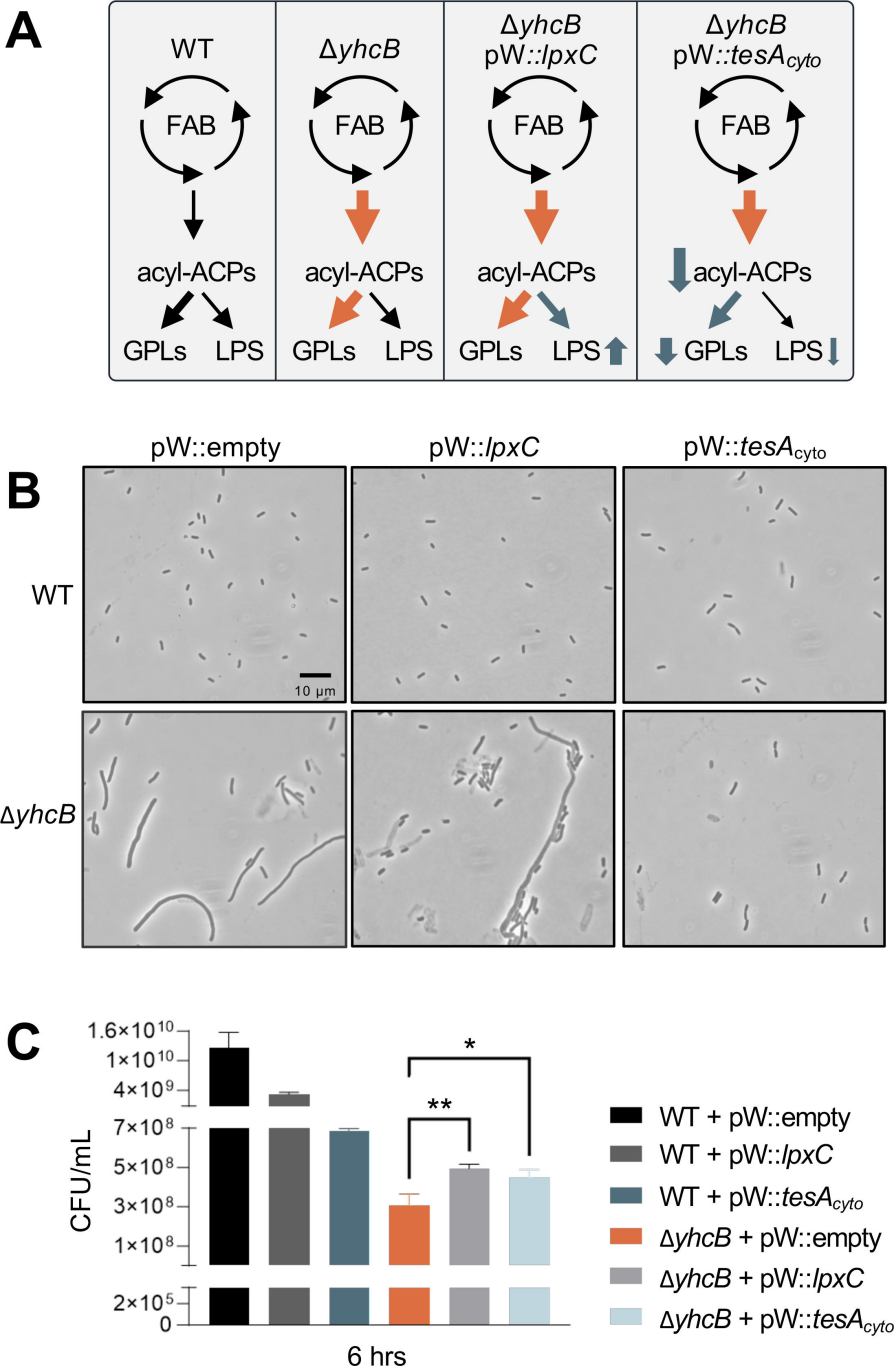
Δ*yhcB* phenotypes originate with excess fatty acids. (**A**) Schematic outlining how overexpression of TesA_cyto_ and LpxC from the low-copy-number, isopropyl β-D-1-thiogalactopyranoside (IPTG)-inducible plasmid pWSKI (pW) is predicted to affect the *yhcB* mutant. In the first panel depicting a wild-type cell, acyl-ACPs generated by FAB disproportionately shunt into GPL synthesis. In the second panel, a *yhcB* mutant has increased flux through FAB and through GPL synthesis, leading to excess GPLs. In the third panel, increased expression of LpxC increases LPS biosynthesis and helps to restore the misbalanced GPL:LPS ratio. In the fourth panel, expression of TesA_cyto_ in the *yhcB* mutant leads to the degradation of FAB end products and reduced GPL and, to a lesser extent, LPS flux. (**B**) Phase-contrast microscopy (×1,000 magnification, 10 µm scale bar) of cells with increased expression of LpxC or a cytoplasmic version of TesA after 6 hours of growth. IPTG was added at initial inoculation, 1 mM IPTG for TesA_cyto_ and 5 µM IPTG for LpxC. (**C**) CFU/mL values determined by serial dilutions of cultures described in panel B. Data shown are representative of biological triplicates. Significance was assessed using two-tailed, unpaired *t*-tests. NS indicates not significant; *, *P*  ≤  0.05; **, *P*  ≤  0.01; ***, *P*  ≤  0.001; ****, *P*  ≤  0.0001.

Although the *yhcB* mutant has excess GPLs and its fitness benefits from reduction of FAB, it was still unclear whether the mutant had excess flux through FAB or through GPL synthesis. Since the products of FAB are disproportionally shunted into GPL synthesis as compared to LPS biogenesis (Fig. 6A), two hypotheses emerged. One, that the *yhcB* mutant exhibits overactive GPL biosynthesis, leading to increased FAB flux due to rapidly used end products. Alternatively, it is FAB that is hyperactive in Δ*yhcB,* and the excess products feed into GPL biosynthesis, resulting in increased GPLs. In short, the origin of the problem could be dysregulated GPL synthesis or dysregulated FAB. To better understand whether the Δ*yhcB* phenotypes were caused by overactive GPL synthesis or overactive FAB, we assessed how manipulation of LPS biogenesis and acyl-ACP degradation would affect the *yhcB* mutant.

LpxC catalyzes the first committed step of LPS biosynthesis, and, for this reason, the cellular levels of enzyme are tightly controlled. We expressed *lpxC* using the low-copy-number, isopropyl β-D-1-thiogalactopyranoside (IPTG)-inducible vector pWSKI (pW in Fig. 6) (35). As previously reported (36), increasing LpxC levels in a wild-type cell was detrimental to fitness as determined by CFU counts after 6 hours of growth. Much like what we saw with our *mla* suppressors, increasing LpxC in the *yhcB* mutant was a weak suppressor that rescued growth but not cell morphology (Fig. 6). TesA is a periplasmic esterase that can act on a variety of acyl-CoA and acyl-ACP molecules; truncation of the gene results in localization of TesA in the cytoplasm (TesA_cyto_) where it can still function and has been demonstrated to generate free fatty acids (37–40). Plasmid expression of TesA_cyto_ in wild-type W3110 resulted in reduced CFU counts at hour 6. Expression of TesA_cyto_ in the *yhcB* mutant, however, strongly suppressed the filamentous morphology and growth defect, suggesting that the degradation of acyl-CoA and acyl-ACP molecules in the cytoplasm is beneficial to cells lacking YhcB (Fig. 6B and C).

These data suggest that increased LPS can restore the asymmetry in the OM and rescue cell growth, but increased LPS does not alleviate the original issue of excess lipids. However, degrading acyl-ACP molecules arising from FAB is highly beneficial in the absence of YhcB. It is only by modulating fatty acids and not LPS synthesis that all *yhcB* phenotypes can be fixed, suggesting that FAB flux in the *yhcB* mutant is abnormally increased.

## DISCUSSION

*E. coli* YhcB has been suggested to play a role in cell division, peptidoglycan synthesis, and even LPS regulation (11, 13, 41). Based on a previous Tn-Seq screen, Goodall and co-authors (13) predicted that *yhcB* may have a synthetically lethal phenotype with 87 genes, with the vast majority of these genes associated with cell envelope biogenesis. Although this work revealed interesting genetic interactions, cells lacking YhcB are both pleiomorphic and show a drastic reduction in bacterial fitness. Therefore, it is not unexpected that the disruption of additional cell envelope pathways would cause cell death, and these synthetic phenotypes may not necessarily indicate a direct relationship with YhcB. Here, we have shown that modulating lipids in the cell directly benefit a *yhcB* mutant and have provided data that link YhcB to FAB.

Through a short-term evolution experiment, we found that the loss of the Mla system can weakly suppress the Δ*yhcB* growth defect but cannot rescue the original defect (demonstrated by the unchanged filamentation). Goodall et al. (13) also reported that any disruption of the Mla system rescued both SDS-EDTA and vancomycin sensitivity in the *yhcB* mutant, though they did not report any cell morphology changes. When Mla is disrupted, GPLs accumulate in the outer leaflet of the OM, leading to increased PldA activity. We show here that rescue of the *yhcB* growth defect conferred by the loss of the Mla system is directly tied to PldA activity (Fig. 3). PldA cleaves GPLs, the by-products of which stimulate LPS biosynthesis. These results also indicate that the defect is not related to a deficiency of GPL transport to the OM since PldA-mediated rescue would only occur if there were too many GPLs in the outer leaflet of the OM. This dependency on PldA is also supported by our finding that the *yhcB* mutant synthesizes an increased number of GPLs (Fig. 4).

Although increasing degradation of mislocalized GPLs and signaling to increase LPS via PldA prohibit the growth defect, these mutations were eventually overtaken by mutations in FAB and GPL synthesis in *yhcB* mutants during a short-term evolution experiment. Of the three populations, the one harboring a mutation in *cdsA* never recovered wild-type cell morphology (Fig. 2B and Fig. S2), suggesting that the defect in the *yhcB* mutant is further upstream. It is interesting to note that these suppressor screens did not uncover any mutations in peptidoglycan or LPS biosynthesis pathways or proteins specific to cell division. Instead, it was mutations in the genes encoding the various subunits of the ACC complex, AccA and AccD, that recovered cellular fitness. The *accA* mutation not only suppressed morphological defects but also stabilized the GPL:lipid A (LPS) ratio within the cell envelope, presumably by reducing flux through FAB (Fig. 4; Fig. S4A and Fig. S5). However, a recent publication by Som and Reddy (42) exploring the crosstalk between FA and peptidoglycan synthesis may explain the apparent peptidoglycan and cell division defects suggested by the filamentous *yhcB* cell. This work demonstrated that MepS, a major endopeptidase involved in peptidoglycan growth, is stabilized in genetic backgrounds with more fatty acid synthesis, including a *yhcB* mutant. These results not only demonstrate a reasonable pathway through which peptidoglycan synthesis responds to the availability of fatty acids, these data also suggest that due to the increased flux through FAB in the absence of YhcB, abundance of fatty acids leads to a concomitant increase in peptidoglycan synthesis, resulting in the filamentous cells.

These genetic findings were recapitulated using a chemical biology approach by inhibiting FAB via cerulenin (Fig. 5). Noga and co-authors (43) present mass spectrometry data that during growth inhibition by cerulenin, longer-chain acyl-ACPs that are preferred substrates for GPLs (C16 and C18 acyl chains) drop rapidly, while levels of acyl-ACPs preferred for LPS increase (C12 and C14 acyl chains). Because cerulenin is an irreversible inhibitor, overall FAB flux would dramatically reduce. Thus, GPL synthesis would decrease at a faster rate compared to LPS synthesis, restoring the balance of OM lipids and the fitness of cells lacking YhcB. The phenotypes of the *yhcB* mutant appear specific to stationary phase, and the presence of cerulenin strongly suppresses both cell morphology and growth defects (Fig. 5A). Fatty acid biosynthesis slows during stationary phase in a normal cell, so perhaps YhcB is involved in reducing flux through the system during this transition. Supporting this hypothesis, two of the Δ*yhcB* populations in our short-term evolution experiment developed mutations in *obgE,* which encodes a GTPase involved in the stringent stress response (44).

Expression of the thioesterase TesA in the cytoplasm strongly suppresses the growth defect and cell morphology in the *yhcB* mutant (Fig. 6). It has been demonstrated that expression of TesA_cyto_ results in an approximately threefold increase in free fatty acids (FFAs) during stationary phase, with excess FFAs even being found in the culture medium. Although the specific role for TesA is still unidentified, analysis of the FFAs generated by TesA_cyto_ activity indicates a substrate preference for long-chain fatty acids such as C16 and C18, which act as acyl donors for GPLs. Some cleavage of acyl donors preferred for LPS synthesis was detected, though in lower amounts compared to the long-chain fatty acids (37). In the *yhcB* mutant, we hypothesize that TesA_cyto_ is degrading the excess acyl-ACPs with preference for acyl donors destined for GPL synthesis, restoring overall fitness. Supporting our work, expression of TesA_cyto_ also rectifies phenotypes associated with overexpression of the regulator FadR which results in increased FAB and GPL synthesis similar to Δ*yhcB* (20). In both scenarios, these results suggest that TesA_cyto_ is generating FFAs from the excess acyl-ACP pool, which can then be degraded through β-oxidation and/or transported into the culture medium through mechanisms that have not been identified.

We expected that if the problem were solely excess GPL flux, then increasing LPS to rebalance lipids in the cell would be beneficial. Indeed, we saw that overexpression of *lpxC* in the *yhcB* mutant mediated the growth defect (Fig. 6C), similar to the PldA-mediated, weak suppression seen with Mla system inactivation (Fig. 3). In support of this finding, Wieczorek et al. (41) demonstrate that LpxC protein levels are stabilized in a *yhcB* mutant. Goodall and co-authors (13) also report that a *yhcB ftsH* double mutant is viable without suppressors. FtsH is an essential protease that proteolytically controls the level of LpxC (and therefore LPS) in the cell. These results are likely due to the cell attempting to rebalance the GPL:lipid A (LPS) ratio to compensate for increased GPL synthesis due to acyl-ACP accumulation. We report that increasing LPS alone cannot fully restore the *yhcB* mutant, evidenced by the filamentous cell morphology (Fig. 6B), because the origin of the problem may be excess flux through FAB, not GPLs. In the context of this information, the synthetic lethality with *lpxM* and the synthetic sick phenotype with *clsA* (Fig. 1) is clear because both of these mutations impact LPS levels and transport (7). Increasing LPS alone cannot fix the *yhcB* cell morphology phenotype, but degradation of acyl-ACPs in the cytoplasm can. These results make sense with the conclusion that FAB is dysregulated and that flux through the biosynthetic pathway is aberrantly increased when YhcB is absent.

However, it is difficult to separate GPL and FAB flux due to the connectivity of these two pathways. Increased FAB flux leads to increased GPL flux, and the opposite is also true (20, 45). For example, increased expression of FadR results in increased production of enzymes involved in FAB, leading to elevated FAB flux and more GPLs (20, 46). Whereas increasing activity of the enzyme that catalyzes the rate-controlling step in GPL synthesis, PlsB, lowers the pool of long-chain ACP species and reduces feedback inhibition of the ACC complex. This elevated activity thereby increases FAB flux in response to raised GPL flux (45). Given the tight connection between the two pathways, experimentally modulating one without impacting the other is impossible. The mutations that evolved in the *yhcB* populations were not in *plsB* but instead in genes encoding subunits of the ACC complex that together determine the rate-controlling step for FAB. The context of these mutations suggests that it is FAB, not GPL synthesis, that is aberrantly increased in the *yhcB* mutant.

The ACC complex (comprised AccABCD) initiates FAB by converting acetyl-CoA to malonyl-CoA in two half-reactions. In the first, AccC carboxylates the biotin attached to AccB (using Mg^2+^ ions as cofactor and ATP), and in the second, AccA and AccD transfer the carboxyl group from biotin to acetyl-CoA, generating malonyl-CoA (47). Thorough understanding of the regulation of the ACC complex has remained elusive, but limited *in vitro* evidence indicates that acyl-ACP molecules (C6 to C20) can inhibit the complex (48, 49). Still, these data have the caveat that the kinetic assays were performed with both holo- and apo-AccB present (biotinylated and unbiotinylated), the latter of which is chemically inert and could prevent ACC complex activity (50). The delicate balance of energy consumption and the need for membrane lipid demands tight control of FAB in bacteria. Here, we have presented evidence that a *yhcB* mutant has lost regulation of this system to deleterious effects. That the best and most repeated response to the loss of YhcB is to mutate any of the subunits of the essential ACC complex suggests that YhcB may play a role in throttling FAB, perhaps at the initiating enzymatic step. Several groups (11, 16, 41) have published data from protein-protein interaction experiments, and only one (41) has reported interactions with phospholipid and fatty acid biosynthesis enzymes (PssA, AccD, and FabBFHY), though direct analysis is required, given the complexity and remaining unknowns of these potential interactions. It is an interesting hypothesis that YhcB may be regulating FAB, through direct or indirect means, and merits further investigation.

## MATERIALS AND METHODS

### Strains and growth conditions

All strains, plasmids, and primers used in this study are provided in Data Set S1 in supplemental material. Unless otherwise stated, strains were grown in lysogeny broth or on LB agar at 37°C. Where indicated, growth media were supplemented with ampicillin (100 µg/mL), kanamycin (30 µg/mL), vancomycin (50 µg/mL), cerulenin (60 µg/mL), L-arabinose (0.05% or 0.1% [wt/vol]) as indicated in figure legends, D-glucose (0.2% glucose [wt/vol]), and/or IPTG (1 mM or 5 µM) as indicated in figure legends. Growth curves were performed both in 0.2 mL LB using the BioTek Epoch 2 plate reader in a polystyrene 96-well plate and in 5 mL LB in glass test tubes.

### Strain construction

Chromosomal deletions were generated in the *E. coli* K-12 strain W3110 via generalized transduction and the Keio collection (21). The flippase recognition target-flanked kanamycin cassette was removed by flippase site-specific recombination as previously described (51). A plasmid, pCP20, which expresses the flippase from a temperature-sensitive promoter, was transformed into the strains possessing the kanamycin resistance cassette. Transformants were then grown at 30°C overnight and then passaged at 37°C. Colonies were screened for loss of both the kanamycin and ampicillin resistance, and the removal of the kanamycin cassette was confirmed via PCR amplification of the appropriate region (51). The strain *lpxM yhcB*::kan + P_ara_::*yhcB* was maintained in the presence of arabinose unless otherwise stated. The mutant *acc* alleles were transferred into relevant strains by linking the mutant allele with a nearby kanamycin cassette. To link these genetic elements, a nearby gene (*yaeR* for *accA* and *purF* for *accD*) with a predicted unrelated function was deleted using generalized transduction, and phage lysate was made from the resulting strain. This lysate was then used to transfer the mutant *acc* alleles with high frequency by selecting for kanamycin resistance in the resulting recombinants. Colonies were then PCR verified for the deletion of *yaeR* or *purF,* and then *accA* or *accD* was Sanger sequenced to verify the point mutation (Data Set S1).

### Scanning electron microscopy

Cultures were grown to OD = 0.5 in 5 mL LB and harvested by centrifugation. Cell pellets were fixed in 2% glutaraldehyde 0.1 M cacodylate buffer for 2 hours at room temperature. Cells were then washed in 0.1 M cacodylate buffer and post-fixed in 1% OsO_4_ 0.1 M cacodylate buffer for 1 hour at room temperature. After washing, samples were dehydrated in an ethanol series (25%, 50%, 75%, and 100% ethanol) for 10 minutes per step. Samples were then placed on poly-L-lysine-coated glass cover slips and critical point dried. Finally, samples were mounted on aluminum SEM stubs and sputter coated with gold-palladium. Images were taken using a Teneo FE-SEM.

### Selecting for Δ*yhcB* suppressors

Suppressors that restored viability of the synthetically lethal *yhcB lpxM* double mutant were obtained via large-scale transductions. Independent cultures of *yhcB* and *lpxM* single mutants were used for separate transductions. Resulting colonies were screened internally and externally via PCR for *yhcB* and *lpxM* and whole-genome sequenced (Data Set S1). Suppressors that rescued formation of cellular debris in the single *yhcB* mutant were obtained from a short-term evolution experiment. Three independent colonies were inoculated in 5 mL LB and passaged daily into fresh 5 mL LB. Aliquots were sent for whole-genome sequencing upon visible resolution of cellular debris (day 7), and additional aliquots were sent after 30 days of passaging. Analysis of the whole-genome sequencing for both suppressor experiments was performed using CLC Genomics Workbench (QIAGEN) and the *E. coli* W3110 genome (GenBank accession number NC_007779).

### Phase-contrast microscopy and cell measurements

Cultures were grown for 5 hours and spotted on 3% agarose pads. Images were captured with the Olympus CX43 equipped with an Olympus Infinity 355 camera in phase contrast. Measurements were performed on >5 fields of view and 298 cells per strain with MicrobeJ and analyzed in GraphPad Prism 10. In the case of the *yhcB* mutant, manual measurements were required for the extremely filamentous cells that were not detectable by MicrobeJ. These cells (<20 cells) were manually measured using the segmented line tool function in ImageJ. A one-way analysis of variance test was performed with Brown-Forsythe and Welch tests, assuming that standard deviations were not equal, and significant differences were assessed using a Games-Howell test.

### Spot plates

Cultures were normalized to an OD = 0.05 and grown in 5 mL LB at 37°C. At each time point, cultures were serially diluted and spotted (5 µL) on LB agar. Plates were incubated overnight at 37°C. A two-tailed, unpaired *t*-test was performed to determine significant differences between samples at specific time points.

### Lipid extractions and visualization

Strains were normalized to an OD of 0.05 in 5 mL LB containing 2.5 µCi/mL of inorganic radioactive phosphate (^32^P_i_) and grown at 37°C for 6 hours. At hour 6, cells were harvested by centrifugation, and total lipids were isolated as previously described (18). Briefly, cell pellets were washed once with 1× PBS, resuspended in a solution of 50 mM sodium acetate (pH 4.35) containing 1% SDS, and incubated at 100°C for 30 minutes. This incubation hydrolyzes the lipid A domain from the polysaccharide portion of LPS and has no effect on GPL structure. The lipid A and GPL species were isolated via Bligh-Dyer extraction, and the lipids were separated using TLC in a solvent system of chloroform, pyridine, 88% formic acid, and water (50:50:16:5, vol/vol). Lipids were visualized by phosphorimaging analysis using the Amersham Typhoon Biomolecular Imager (Cytiva), and densitometry was performed to determine the GPL:lipid A ratios. Images shown in figures have been adjusted for visibility of all lipid species, and an unsaturated image was used for all densitometry calculations. A two-tailed, unpaired *t*-test was performed to determine significant differences between samples.

### LPS quantification

Strains were grown for 6 hours from a starting OD of 0.5 and harvested by centrifugation. Samples were normalized by protein concentration, and the cell pellet was resuspended in lithium dodecyl sulfate loading buffer and treated with proteinase K. Samples were analyzed by SDS-PAGE using a 4%–12% Bis-Tris gel (Fisher). Following the manufacturer’s instructions, LPS was stained using the Pro-Q Emerald Kit. Gels were visualized using the Bio-Rad ChemiDoc MP Imaging System, and relative LPS values were calculated using densitometry of the stained bands. A two-tailed, unpaired *t*-test was performed to determine significant differences between samples.

## SUPPLEMENTAL MATERIAL

Data Set S1 mbio.00790-24-s0001.xlsx Strains, plasmids, and primers.

Supplemental Figures mbio.00790-24-s0002.docx Figures S1 to S5.

ASM does not own the copyrights to Supplemental Material that may be linked to, or accessed through, an article. The authors have granted ASM a non-exclusive, world-wide license to publish the Supplemental Material files. Please contact the corresponding author directly for reuse.
