# Supplementary material for: Loss of YhcB results in overactive fatty acid biosynthesis
Source: mBio. 2024 May 14;15(6):e00790-24. doi: 10.1128/mbio.00790-24 (PMC11237625; doi:10.1128/mbio.00790-24)
Supplement: Supplemental Figures — Figures S1 to S5. [file mbio.00790-24-s0002.docx]

**Supplemental material** supporting “Loss of YhcB results in overactive fatty acid biosynthesis” by HM Stanley and MS Trent.

**
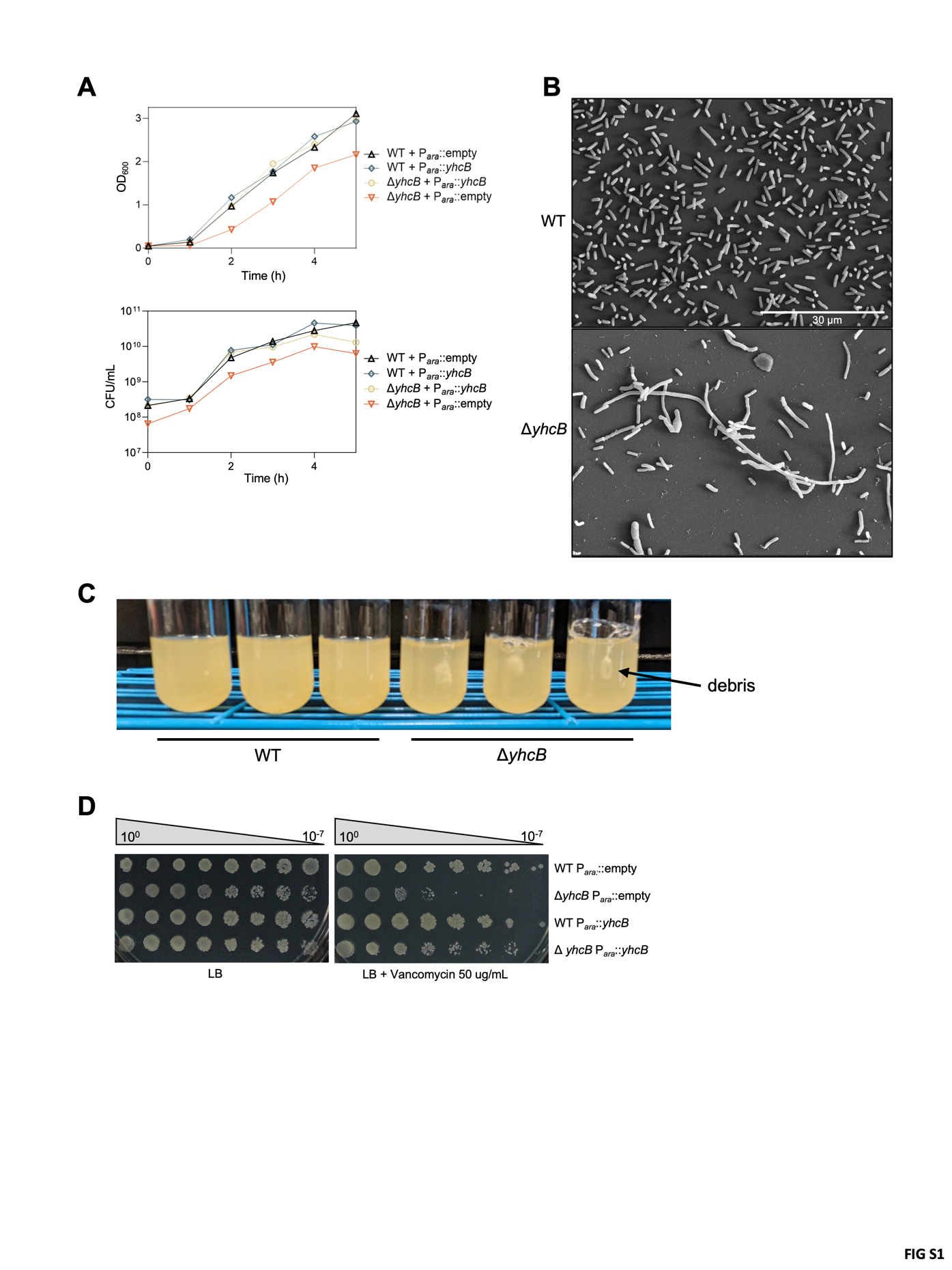
**

**FIG S1 Altered YhcB levels affect growth, cell morphology, and vancomycin sensitivity.** (A) Growth of the indicated strains was measured in LB every hour by OD_600_ and by serial dilutions to calculate CFU/mL. 0.05% arabinose was used to induce plasmid gene expression. Data shown is representative of biological triplicates. (B) Cultures of wild type W3110 and the *yhcB* mutant (OD_600_=0.5) were subjected to scanning electron microscopy (SEM). Micrographs are shown at 10,000x (Scale bar, 30 µm). (C) Photograph of wild type and Δ*yhcB* after 24 hours of growth at 37˚C when cell debris is visible by eye in the Δ*yhcB* culture, marked by the black arrow. (D) Efficiency of plating assays of the indicated strains were performed by normalizing cultures to OD 1.0 and serially diluting on LB and LB containing 50 µg/mL of vancomycin before overnight incubation at 37˚C. Data shown is representative of biological triplicates.

**
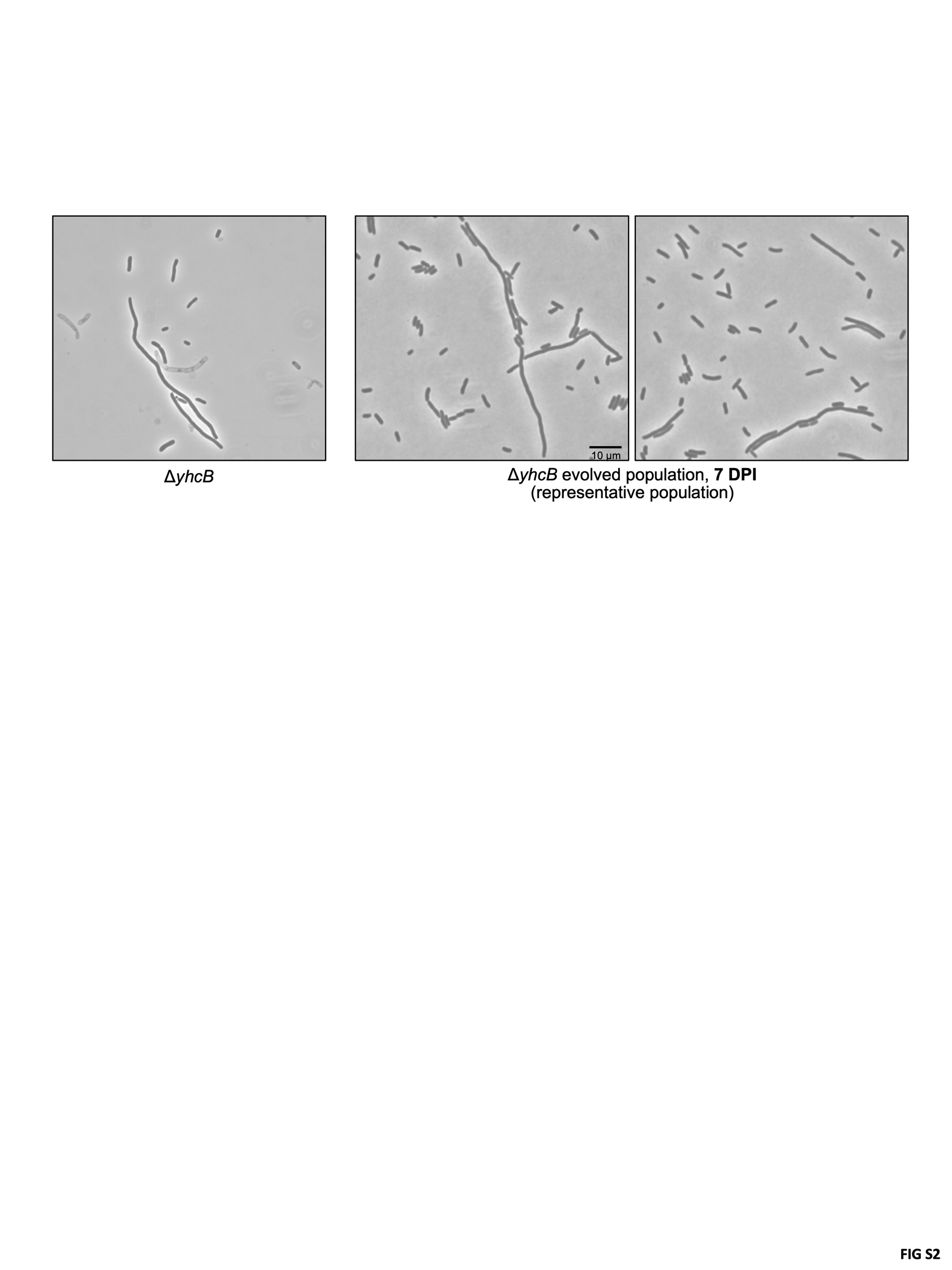
**

**FIG S2 Evolved populations of *yhcB* at time of debris resolution are still filamentous.** Phase contract microscopy of an Δ*yhcB* evolved population at 7 DPI when cell debris was resolved, 1000x (scale bar is 10 µm). DPI – days post inoculation

**
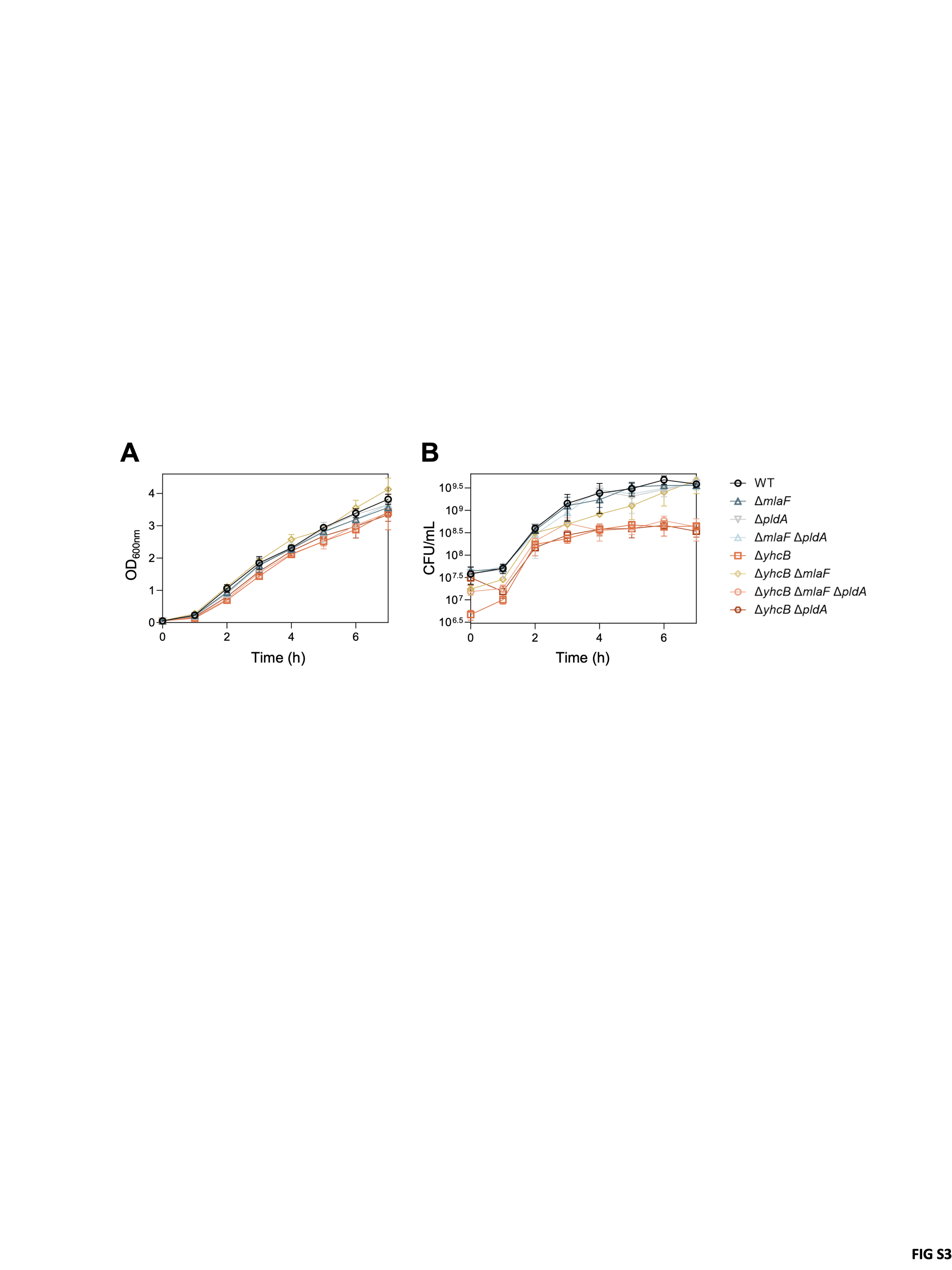
**

**FIG S3 Loss of MlaF and PldA have no effect on wild type growth.** Growth curves were performed with a starting OD of 0.05 in 5 mL LB and measured by both OD_600_ (Panel A) and by serial dilutions (Panel B) to calculate CFU/mL. Data shown is representative of biological triplicates.

**
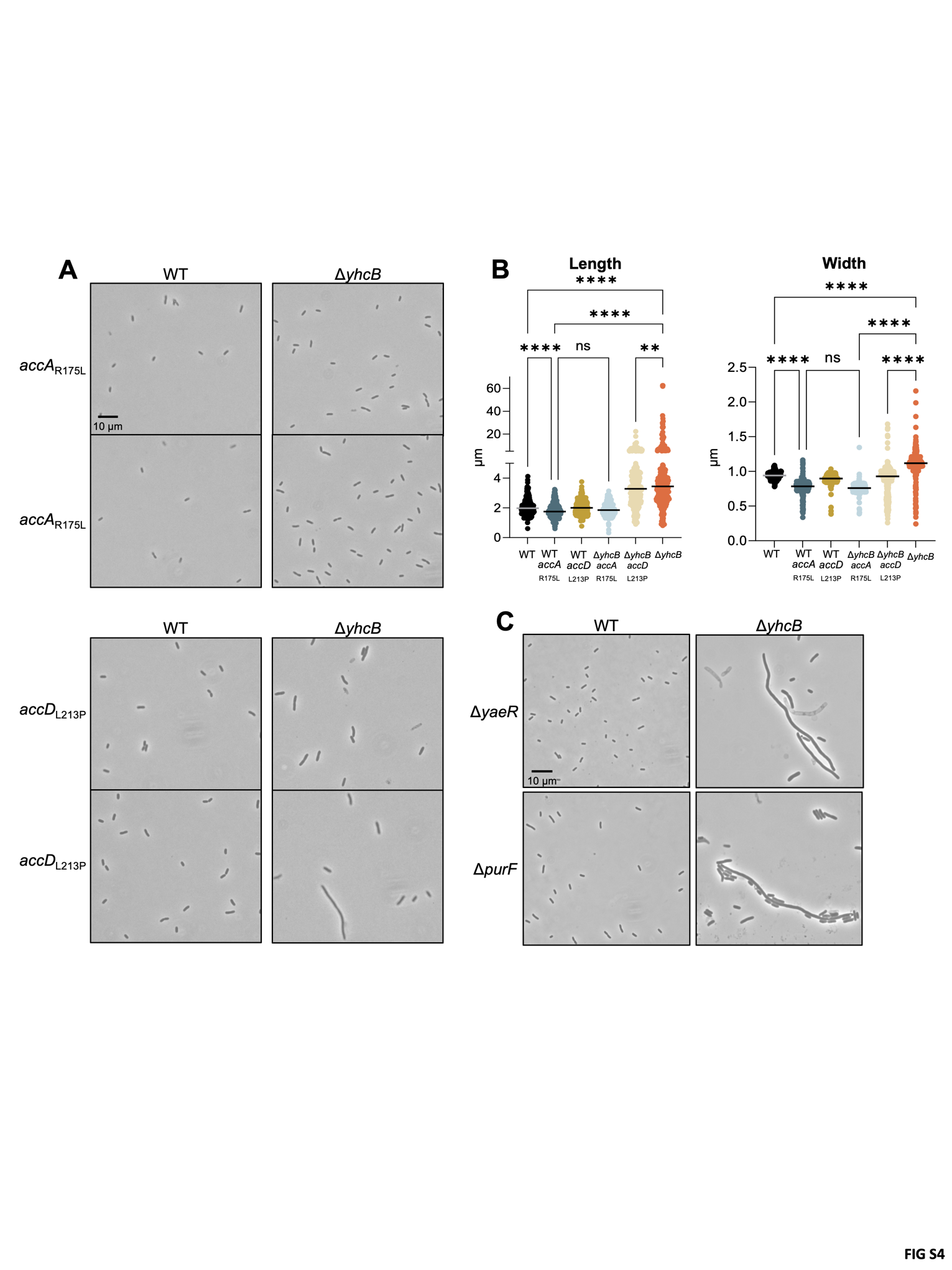
**

**FIG S4 Microscopy analysis of *acc* alleles in wild type and the *yhcB* mutant**. (A) Phase-contrast microscopy at x1000 magnification with a 10-μm scale bar of wild type and the *yhcB* mutant with alleles of *acc* (*accA*_R175K_ and *accD*_L213P_) replacing the wild-type copy on the chromosome. (B) Cell size analysis was performed on 298 cells per strain with MicrobeJ, with manual measurements of extremely long cells performed in ImageJ, and measurements were assessed through a one-way analysis of variance (ANOVA) test with Brown-Forsythe and Welch tests assuming that standard deviations were not equal. Significant differences were assessed using a Games-Howell test. NS indicates not significant; *, *P*≤ 0.05; **, *P*≤ 0.01; ***, *P*≤ 0.001; ****, *P*≤ 0.0001. (C) Phase-contrast microscopy at x1000 magnification with a 10-μm scale bar of wild type and the *yhcB* mutant with *yaeR* or *purF* deleted from the chromosome.

**
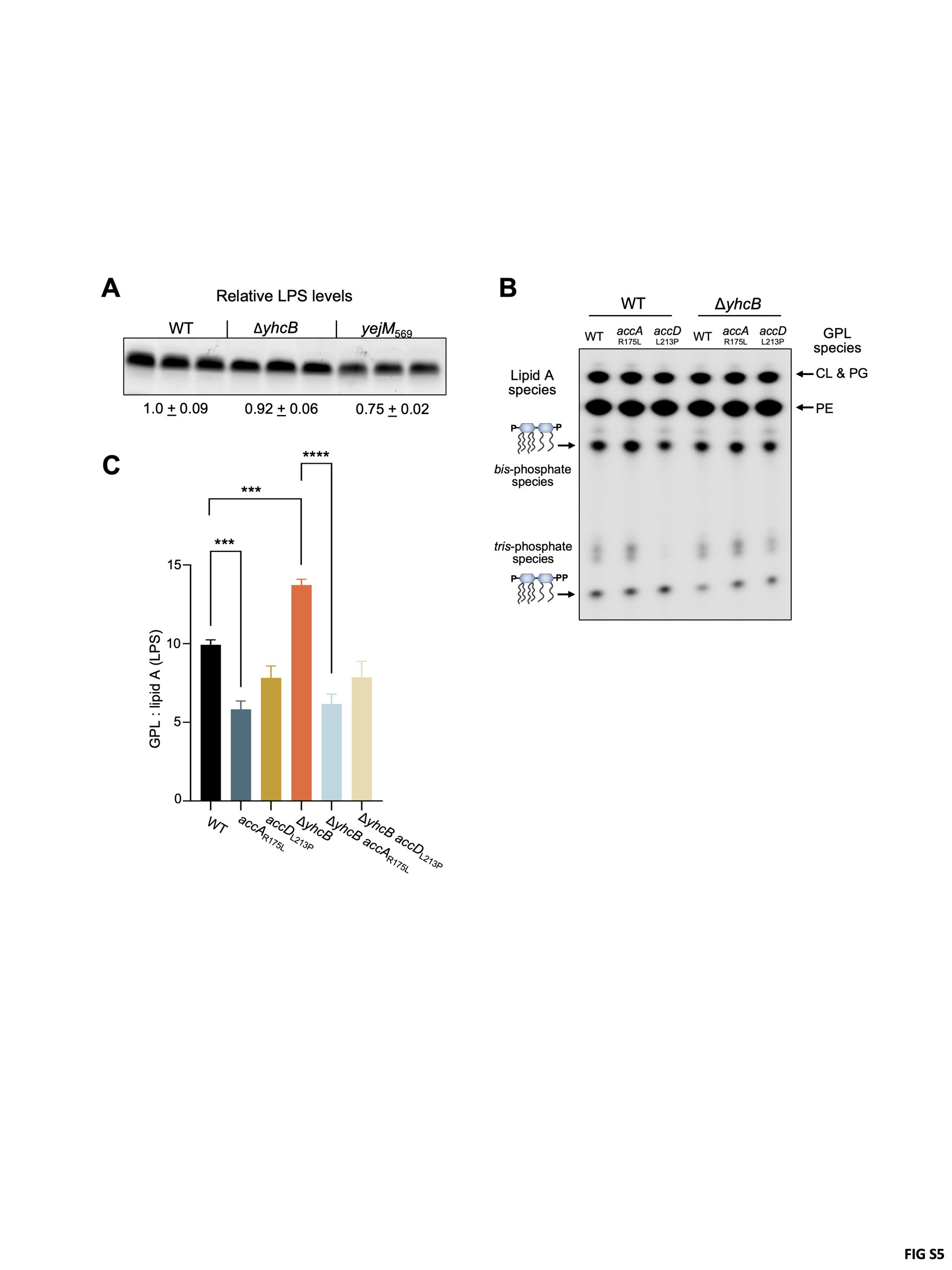
**

**FIG S5** **Evaluation of LPS and GPL levels in Δ*yhcB* and strains harboring mutant alleles of *accA/D*.** (A) LPS levels, relative to wild type, were determined by SDS-PAGE of proteinase K-treated cell lysates of the indicated strains. Samples were run on a 4-12% gradient Bis-Tris gel, and the LPS was stained using ProQ Emerald Green and quantified by densitometry. Samples are biological replicates and were grown for 6 hours from starting OD=0.05. (B) TLC of total ^32^P-labeled lipids (both lipid A and GPLs) of either wild type or the *yhcB* mutant harboring different *accA/D* alleles. Image shown has been adjusted for visibility of all lipid species; an unsaturated image was used for densitometry calculations. (C) Bar graphs representing the GPL:lipid A (LPS) ratio calculated from TLC data in panel B. Data shown is representative of biological triplicates. Significance was assessed using two-tailed, unpaired T-tests. NS indicates not significant; *, *P*≤ 0.05; **, *P*≤ 0.01; ***, *P*≤ 0.001; ****, *P*≤ 0.0001.

**DataSet S1 (separate file). Strains, plasmids, and primers used in this manuscript.** Tab 1) Strains and plasmids used in this study. Strains that were assessed by whole genome sequencing are indicated. Tab 2) Primers used in this study**.**
